# Associations between childhood risk factors and alcohol treatment outcomes in adolescence

**DOI:** 10.1093/alcalc/agaf081

**Published:** 2026-01-15

**Authors:** Mica Komarnyckyj, Dylan Mangan, Karen P Hayhurst, Stephen J Kaar, Stefan Jahr, Andrew Jones

**Affiliations:** Manchester Biomedical Research Centre, Division of Psychology & Mental Health, University of Manchester, Oxford Rd, Manchester M13 9PL, United Kingdom; National Drug Evidence Centre, Division of Population Health, Health Service Research and Primary Care, University of Manchester, Manchester M13 9PL, United Kingdom; National Drug Evidence Centre, Division of Population Health, Health Service Research and Primary Care, University of Manchester, Manchester M13 9PL, United Kingdom; Division of Psychology & Mental Health, University of Manchester, Oxford Rd, Manchester M13 9PL, United Kingdom; Greater Manchester Mental Health NHS Foundation Trust, Addictions Services, Chapman-Barker Unit, Manchester M25 3BL, United Kingdom; Royal College of Psychiatrists Child and Adolescent Mental Health, Faculty Executive Committee, London, United Kingdom; Department of Health and Social Care, Office for Health Improvement and Disparities (OHID), London, United Kingdom; National Drug Evidence Centre, Division of Population Health, Health Service Research and Primary Care, University of Manchester, Manchester M13 9PL, United Kingdom

**Keywords:** adolescence, alcohol, addiction, predictors, binge drinking, adverse childhood experiences (ACEs)

## Abstract

**Background:**

Early onset alcohol use, mental health problems, familial alcohol use, and adverse childhood experiences (ACEs) increase the likelihood of persistent alcohol-use disorders later in life. This study aimed to determine the relative influence of such risk factors when predicting adolescent alcohol treatment outcomes, an area overlooked in prior research.

**Methods:**

Retrospective cross-sectional analysis using the National Drug Treatment Monitoring System, incorporating data from all publicly funded community alcohol services in England. The study included adolescents (aged 11–17) whose alcohol treatment took place between 1 April 2018 and 31 March 2023 (*n* = 2621). Logistic regression models tested for associations between predictors and key outcomes, including treatment non-completion and being non-abstinent at treatment completion. Predictors included demographics, treatment information, alcohol use, ACEs, care status, psychological, and socioeconomic factors.

**Results:**

Significant predictors of not completing treatment: increased age; increased monthly drinking days; year of exit 2020–2021; Not in Education, Employment or Training (NEET) status and being placed on a child protection plan. NEET adolescents had double the incomplete treatment rate compared to the general cohort. Significant predictors of non-abstinent completion: increased age, monthly drinking days, units per drinking day, mental health treatment need, early onset use, affected by others’ substance use, and illicit substance use.

**Conclusions:**

Adolescents with higher alcohol or illicit substance use at treatment start, NEET status and/or child protection plan (care status indicating prior ACE exposure) have worse alcohol treatment outcomes. These groups are highlighted for tailored interventions which consider psychological and environmental challenges adolescents may be experiencing.

## Teaser

Adolescents with higher alcohol use at treatment start tend to have poorer alcohol treatment outcomes in England. NEET status and being placed on a child protection plan also increased the risk of treatment dropout. This highlights the need for trauma-informed and socially responsive interventions and for policy commitments that address the structural determinants of poor treatment engagement.

## Introduction

Childhood risk factors increase a person’s vulnerability for developing alcohol or substance use problems later in life (Rozin and Zagonel 2012, Nawi et al. 2021). Risks include early onset use (Rozin and Zagonel 2012, Donoghue et al. 2017, Yuen et al. 2020), mental health problems (Swendsen et al. 2010, Puddephatt et al. 2022), and environmental exposures such as peer substance use (Rozin and Zagonel 2012, Yuen et al. 2020) and adverse childhood experiences (ACEs) (e.g. abuse, neglect, and familial substance misuse) (Rozin and Zagonel 2012, Yuen et al. 2020, Leza et al. 2021). An understanding of how these risks are associated with treatment outcomes could aid the development of targeted interventions and personalized care, with potential for improving treatment services (Camenga et al. 2022). The influence of childhood risk factors on alcohol and substance treatment outcomes, however, remains largely unexplored (Adamson et al. 2009, Hong et al. 2024, Ingesson-Hammarberg et al. 2024, Wells et al. 2024).

Prior studies have focussed on the use of routinely collected data to model how current demographic, alcohol/substance use, psychopathology, and personality traits predict treatment outcomes. Regression and machine-learning algorithms have been used to analyse large population-level datasets (Adamson et al. 2009, Hong et al. 2024, Ingesson-Hammarberg et al. 2024, Wells et al. 2024). For adults within alcohol treatment, lower drinking levels at treatment start and being female were associated with improved drinking control (Ingesson-Hammarberg et al. 2024), whereas greater alcohol dependence severity and psychopathology consistently predict poorer outcomes (e.g. non-abstinence, reduced engagement) (Adamson et al. 2009). Increased complexity of behavioural needs and involvement with the criminal justice system have been linked to early exit from adult substance use treatment programmes (Hong et al. 2024). Among adolescents (aged 10–17 years), substance use treatment completion rates were lower for those with polysubstance use and housing insecurity (Wells et al. 2024).

Within this literature, an evidence gap exists of how childhood risk factors predict treatment outcomes (Adamson et al. 2009, Hong et al. 2024, Ingesson-Hammarberg et al. 2024, Wells et al. 2024). We identified several studies explicitly modelling their impact on adolescent substance use treatment outcomes; the results of these studies provide an inconclusive picture, and none of the studies relate to an English population (Titus et al. 2003, Yildiz et al. 2020, Coffman et al. 2023, Eddie et al. 2024). Recent U.S. studies report no associations between post-traumatic stress disorder and treatment engagement across three age groups (12–17, 18–25, 26+) (Eddie et al. 2024) and no associations between traumatic stress symptoms and substance use treatment outcomes in victimized adolescents (Coffman et al. 2023).

In contrast, an earlier U.S. study found severity of victimization, including physical, sexual and/or emotional abuse, predicted 3-month post-discharge substance use treatment outcomes (Titus et al. 2003). Furthermore, a Turkish study examining the relationship between ACEs (abuse and neglect) and substance use treatment completion among adolescents (aged 11–17) (Yildiz et al. 2020) found that psychiatric comorbidities (including depression, attention deficit hyperactivity disorder, and dissociative amnesia) were associated with early exit from treatment. The experience of childhood emotional neglect and/or abuse was also associated with non-completion of an inpatient treatment programme (Yildiz et al. 2020).

Previous studies of adolescents (Titus et al. 2003, Yildiz et al. 2020, Coffman et al. 2023, Eddie et al. 2024, Wells et al. 2024) have modelled outcomes for combined alcohol and substance use treatment; none have focussed specifically on alcohol. As one of the most widely used and problematic substances for young people (OHID 2024, WHO 2024), alcohol warrants dedicated research to uncover unique risk factors for this group. This is the first study to compare the relative influence of childhood risk factors on outcomes for adolescents who are in treatment solely for problematic alcohol use. Here, we examine the prevalence of childhood risk factors within English alcohol treatment services using the National Drug Treatment Monitoring System (NDTMS), a centralized database which receives monthly input from all publicly funded alcohol and substance misuse treatment services in England (OHID 2023, NHS England 2024). This pre-existing database tracks a wide range of risks for under-18s, including ACEs, care status, socioeconomic, psychological, and behavioural factors. This study aims to uncover specific factors which predict poorer treatment outcomes, such as individuals dropping out before the end of planned treatment.

## Methods

### Data selection

We extracted data from the NDTMS for all alcohol-only treatments (i.e. not receiving treatment for other substances), which were both started and ended between 1 April 2018 and 31 March 2023 by adolescents who were aged 11–17 and treatment naive at treatment start. This period was the most recent available at the time of analysis, where collected data items remained consistent. Adolescents were excluded if: treatment continued for over 24 months, no treatment outcomes profile (TOP) (Supplementary Material 1.2) record was identified for the individual or if data for the previous 28-day alcohol use (days drinking or units consumed per drinking day) at treatment start were missing. The final dataset included 2621 adolescents (Supplementary Material Table S1).

### Variables

All variables were calculated using established, published methods produced by the Office for Health Improvement and Disparities (OHID) (Public Health England 2022, OHID 2023, 2024).

#### Childhood risk factors

Self-reported at treatment start to the treatment provider, based on the adolescent’s current situation, unless otherwise stated. For detailed descriptions of the NDTMS data underlying risk factors, see Supplementary Material 1.4 and 1.5.

- **Psychological factors:** Mental Health Need (either current diagnosis or self-reported to currently be experiencing symptoms); Self-Harm (at any prior time).
- **ACEs:** Witnessing Domestic Abuse (at any prior time); Being affected by Others’ Substance Use (specifically a family/household member); Sexual Exploitation (at any prior time).
- **Care Status—Indicator of prior ACEs:** Child In Need (need for help or protection due to risks to health or development may be experiencing or at risk of ACEs); Child Protection Plan (ongoing risk of significant harm due to ACEs including neglect and/or physical/sexual/emotional abuse (NSPCC 2024), has not been removed from the harmful environment); Looked After Child (under local authority care for over 24 hours due to parental inability to look after the child, child welfare concerns, or criminal charges. Typically has a history of ACEs severe enough that removal was necessary, though the child is no longer in that harmful environment).
- **Behavioural factors:** Early Onset Use (alcohol use initiated <15 years); Anti-Social Behaviour (antisocial behaviour or criminal act on more than 1 occasion in the past 6 months); Illicit Substances (used in the 28 days before treatment start).
- **Social and economic factors:** Not in employment, education or training (NEET); Housing Problem; Pregnant or Parent.

#### Alcohol use

Two self-reported variables were used to characterize adolescent drinking levels in the 28 days before entering treatment:

- The number of days consumed alcohol.
- The mean alcohol units per drinking day.

#### Demographic and treatment information

- Age in years at treatment start.
- Sex (male/female).
- Source of referral to treatment (grouped as: education, youth/criminal justice, social care, self, family and friends, health services, substance misuse services, other).
- Financial year in which each treatment ended.
- Behavioural Disability (self-reported by adolescents based on a lack of control over their feelings or actions).

### Analysis

Logistic regression models (Model 1 and Model 2) were used to test for predictors of treatment outcomes.

The outcomes of interest within Model 1 were treatment exits defined as:

- **Completion:** finished planned treatment (abstinent or non-abstinent) vs.
- **Incomplete:** did not finish planned treatment (i.e. dropped out).

Within Model 2 the completion category was divided into:

- **Abstinent completion:** completed planned treatment and achieved abstinence from alcohol vs.
- **Non-abstinent completion:** completed planned treatment but did not achieve abstinence from alcohol.

For mapping of outcomes onto NDTMS discharge codes (OHID 2023), see Supplementary Material 1.6.

All variables were included in initial models and checked for multicollinearity using the generalized variance inflation factor. ‘Source of Referral’ and ‘Financial Year of Exit’ were dummy coded to permit selection of initial reference groups; in the former ‘Education’ as this was the largest group, in the latter, financial year 2018–2019 was selected to better illustrate change over time from a baseline. Stepwise (both directions) selection was performed, i.e. predictors are removed/added one at a time until further removal/addition of predictors ceases to lower the model’s Bayesian Information Criterion. This produces parsimonious sets of predictors, from which Model 1 and Model 2, along with odds ratios, were calculated.

**Figure 1:**
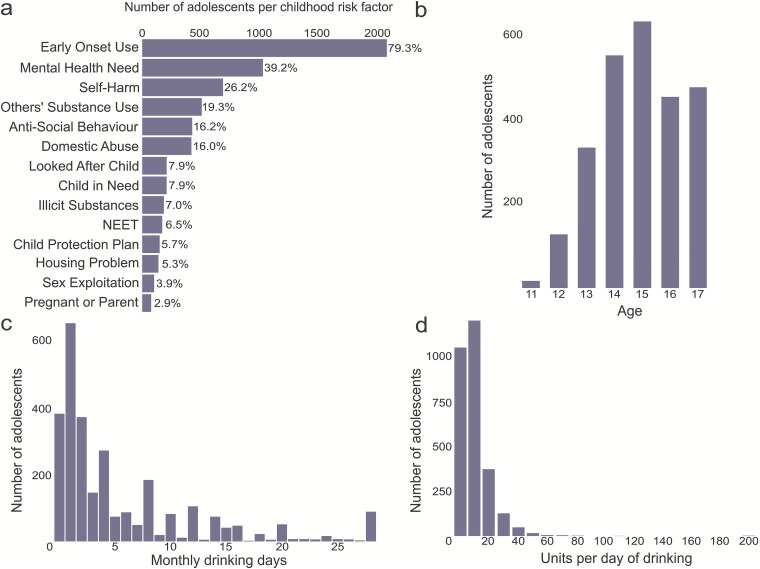
Prevalence of childhood risk factors and alcohol consumption distributions, among English adolescent alcohol treatments. Note: (a) Prevalence of childhood risk factors among adolescents accessing alcohol treatment services in England. (b) Distribution of age across the sample of adolescents accessing alcohol treatment services. (c) Distribution of the number of days alcohol has been consumed in the last 28 days within the cohort. (d) Distribution of the mean number of units of alcohol that were consumed on each drinking day within the last 28 days across the cohort.

Inspection of Hosmer-Lemeshow tests (H-LT) and skewed distributions of age (Fig. 1b), monthly drinking days (Fig. 1c), and units per occasion (Fig. 1d) indicated these predictors should be transformed (age^#^ = age at treatment start squared, Monthly drinking days^*^ = $\sqrt[2]{\mathrm{Monthly}\ \mathrm{Drinking}\ \mathrm{Days}}$, units per drinking occasion^ = $\sqrt[3]{\mathrm{Units}\ \mathrm{per}\ \mathrm{drinking}\ \mathrm{day}}$). To ensure these transformations did not alter predictor selection, collinearity and step were repeated using these variables; the same predictors were selected as presented in the main results. Residuals were used to check for potential problems with outliers and dispersion; no problems were highlighted (Supplementary Figs S1–S4). A significance level of *α* = .05 was used throughout. Data processing software are listed in Supplementary Material 1.7.

## Results

### Cohort descriptives and prevalence of childhood vulnerabilities

A total of 2621 adolescent treatments met the inclusion criteria for the study; 62.6% were females. The median age was 15 years (IQR 14–16) (Table 1) (Fig. 1b). The most common risk factor, reported in 79.3% of treatments, was early onset use, followed by mental health need (39.2%) and previous self-harm (26.2%) (Fig. 1a, Supplementary Table S2).

**Table 1:** Descriptive statistics for age and alcohol consumption, and prevalence of childhood risk factors, split by incomplete and complete treatments

**Variable**	**Whole sample**		**Incomplete treatment group**		**Complete treatment group**	
	**Median**	**IQ range, number (*n*)**	**Median**	**IQ range, number (*n*)**	**Median**	**IQ range, number (*n*)**
Age	15	IQR = (14–16), *n* = 2621	16	IQR = (15–17), *n* = 263	15	IQR = (14–16), *n* = 2358
Monthly drinking days	2	IQR = (1–8), *n* = 2621	6	IQR = (1.5–14), *n* = 263	2	IQR = (1–7), *n* = 2358
Units per occasion	8	IQR = (2–15), *n* = 2621	10	IQR = (5–18.5), *n* = 263	8	IQR = (2–14), *n* = 2358
	**Whole sample**		**Incomplete treatment group**		**Complete treatment group**	
**Childhood risk factor**	***n***	**% of sample**	***n***	**% of sample**	***n***	**% of sample**
Early onset use	2079	79.32	192	9.24	1887	90.76
Mental health need	1026	39.15	130	12.67	896	87.33
Self-harm	687	26.21	87	12.66	600	87.34
Others’ substance use	506	19.31	68	13.44	438	86.56
Anti-social behavior	425	16.22	54	12.71	371	87.29
Witnessing domestic abuse	418	15.95	58	13.88	360	86.12
Looked after child	208	7.94	38	18.27	170	81.73
Child in need	208	7.94	25	12.02	183	87.98
Illicit substances	184	7.02	21	11.41	163	88.59
NEET	170	6.49	44	25.88	126	74.12
Child protection plan	148	5.65	27	18.24	121	81.76
Housing problem	138	5.27	22	15.94	116	84.06
Sex exploitation	101	3.85	9	8.91	92	91.09
Pregnant or parent	76	2.9	10	13.16	66	86.84

### Alcohol use

The median number of days consuming alcohol in the 28 days before treatment entry was 2 (IQR 1–8), with a median number of units consumed per drinking day of 8 (IQR 2–15), roughly equivalent to an 11% ABV. 750 ml bottle of wine (Table 1). With 80% (*n* = 2107) consuming alcohol on fewer than 10 days and 4% (*n* = 101) drinking alcohol on 24 or more days (out of the last 28 days), there were few physically dependent daily/almost daily drinkers in treatment. At-risk drinking behaviours are, however, evident within the cohort, as illustrated by alcohol use distributions in Fig. 1c and 1d.

### Rate of incomplete versus complete treatments

**Figure 2:**
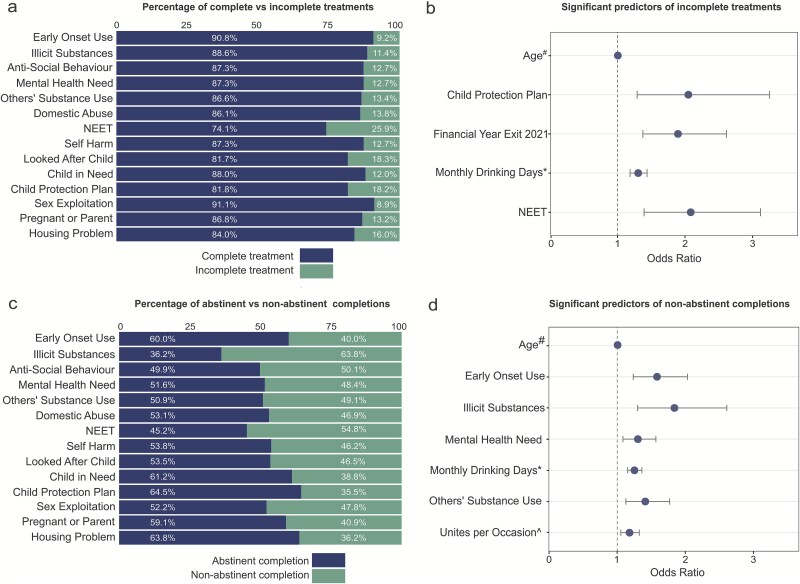
Rates of incomplete and non-abstinent adolescent alcohol treatments alongside odds ratio plots. Note: (a) Complete and incomplete treatment rates by childhood risk factors among adolescents exiting alcohol treatment services in England. (b) Odds ratio plot for logistic regression model 1 showing significant predictors of incomplete alcohol treatments. (c) Non-abstinent and abstinent completion rates by childhood risk factors among adolescents exiting alcohol treatment services in England. (d) Odds ratio plot for logistic regression model 2 showing significant predictors of non-abstinent alcohol treatment completions.

Among treatment exits, 263 (10.3%) were incomplete and 2358 (89.7%) were complete. Figure 2a shows how exits were distributed within treatments flagged with each risk factor. The NEET group had 25.9% incomplete treatments, which was more than double that of the general cohort. Having a child protection plan was associated with an 18.2% incomplete treatment rate and being a looked after child with 18.3%.

### Predictors of incomplete treatments (Model 1)

Model 1 had a H-LT *χ*^2^ = 7.97, (d.f. = 9, *P* = .54). Three risk factors emerged as significant predictors of an incomplete treatment, compared to a complete treatment [i.e. odds ratio (OR) >1] (Table 2 and Fig. 2b): having a financial year of exit of 2020–2021 compared to exiting during any other financial year (OR: 1.90, 95% CI: 1.38–2.61, *P* < .001), having a child protection plan (OR: 2.05, 95% CI: 1.38–2.61, *P* < .01), and being defined as NEET (OR: 2.08, 95% CI: 1.39–3.12, *P* < .001). For initial pre-step Model 1 results, see Supplementary Table S3.

Two transformed predictors also emerged as significant: increased age^#^ (OR: 1.01, 95% CI: 1.00–1.01, *P* < .01) and increased monthly drinking days^*^ (OR: 1.31, 95% CI: 1.19–1.44, *P* < .001). Supplementary Table S4 gives specific examples of these values for real-world increases in the pre-transformed predictors: for a 16-year-old relative to a 14-year-old OR ≈ 1.42, and for eight drinking days per month relative to 1 day OR ≈ 1.63. Supplementary Material 2.2. gives further details on the interpretation of transformed odds ratios.

### Rate of non-abstinent versus abstinent completions

Within the subsample of 2358 treatment completions, 937 individuals (39.7%) were non-abstinent and 1421 (60.3%) abstinent. Figure 2c shows the rates of abstinent and non-abstinent completions by risk factor. Illicit substance use had the highest rate of non-abstinent completions at 63.8%, followed by NEET (54.8%) and anti-social behaviour (50.1%). Supplementary Table S5 shows a growing proportion of non-abstinent completions each financial year, from 35.9% in 2018–2019 to 43.8% in 2022–2023.

### Predictors of non-abstinent treatment completions (Model 2)

Model 2 had a H-LT *χ*^2^ = 9.41, (d.f. = 8, *P* = .31). Four risk factors emerged as significant predictors of a non-abstinent completion, i.e. OR > 1 (Table 3 and Fig. 2d): mental health need (OR: 1.30, 95% CI: 1.08–1.57, *P* < .01); early onset use (OR: 1.59, 95% CI: 1.24–2.03, *P* < .001); being affected by other’s substance use (1.41, 95% CI: 1.13–1.77, *P* < .01); use of illicit substances (OR: 1.84, 95% CI: 1.30–2.61, *P* < .001). For initial pre-step Model 2 results, see Supplementary Table S6.

Three transformed predictors were also significant: increased age^#^ (OR: 1.01, 95% CI: 1.1.01–1.01, *P* < .001); increased monthly drinking days^*^ (OR: 1.25 95% CI: 1.15–1.36, *P* < .001) and increased units per drinking occasion^^^ (OR: 1.18, 95% CI: 1.05–1.32, *P* < .01). Supplementary Table S7 gives examples of these values for real-world increases in the pre-transformed predictors: for a 16-year-old relative to a 14-year-old OR ≈ 1.80, for 7 drinking days per month relative to 1 day OR ≈ 1.45 and for 14 units per drinking occasion relative to 2 units OR ≈ 1.21.

### Evaluating the impact of exclusions due to missing data

Model 1 and Model 2 were also run including individuals who were excluded due to missing data (Supplementary Table S8). In both cases, stepwise selection (Supplementary Tables S9 and S10) yielded the same variables with similar coefficients/odds ratios as in the main analysis (Tables 2 and 3), indicating that these exclusions do not affect our overall findings.

## Discussion

This was the first study to explore the prevalence and relative influence of childhood risk factors on alcohol treatment outcomes in adolescents in England. We found that those with higher alcohol use at treatment start, together with those who were NEET and those placed on child protection, experienced poorer treatment outcomes. Our results highlight how economic disadvantage, and safeguarding risks intersect with alcohol use to influence treatment trajectories of adolescents. Tackling these disparities requires both targeted interventions and wider policy commitments to reduce socioeconomic disadvantage and to embed proactive, trauma-informed approaches in alcohol treatment.

Recent research highlights a long-term reduction in the number of adolescents entering alcohol treatment in England, while the proportion of those entering with high levels of alcohol consumption has increased (Hayhurst et al. 2024). The majority of adolescents in treatment in this study were regular binge drinkers, typically drinking a median of 8 units per occasion, on a median of 2 days per month, where binge drinking is defined as consuming over 6 (female) or 8 (male) units of alcohol in one session (DHSC 2021). While usage patterns do not indicate large numbers of physically dependent drinkers, underreporting of use may have occurred, and NDTMS does not capture clinical assessments on alcohol use disorder (AUD) (e.g. Alcohol Use Disorder Identification Test (AUDIT), DSM-5); therefore, we cannot comment on the prevalence of AUD in this population.

Our modelling supports prior literature: higher alcohol use at treatment start predicts worse treatment outcomes (Adamson et al. 2009). A history of more frequent drinking increased the likelihood of not completing treatment and of non-abstinent completion. Increased units per occasion also raised non-abstinent completion odds. We found that the proportion of non-abstinent completions continues to rise each year. Future work should seek to understand why this is the case, given that abstinence is advised in Government guidelines as the healthiest and safest option for under-18s (NHS England 2022).

Abstinence from alcohol is not a mandated treatment goal within English adolescent services, and treatment providers may focus on harm reduction techniques rather than abstinence, equipping individuals with strategies for safe or controlled drinking. A greater understanding of the barriers to abstinence is, however, key because of the increased risk later in life of AUD for those individuals who are early onset drinkers with hazardous drinking behaviours (Hingson and Zha 2009, Yuen et al. 2020, Tackaberry-Giddens et al. 2023).

We demonstrate that for adolescents, increased age at treatment start predicted worse outcomes; each additional year of age at treatment start increased the likelihood of not completing treatment and completing non-abstinent, especially for those at the higher end of the age distribution. A recent U.S. study found adolescents (aged 12–17) were more likely to engage with substance use treatment than over eighteens. Although substance use disorder can be particularly hard to treat in adolescents, the authors suggest this might be mitigated by the influence of parents (Eddie et al. 2024). Expanding on this, we found that within an under-18 cohort, differences in treatment success also exist. This may be due to reduced parental control and increased autonomy with more opportunities to conceal behaviour in later adolescence.

NEET individuals had the highest rate of incomplete treatments (25.9%)—over double that of the general cohort. Being NEET was also a significant predictor of incomplete treatments. Risk factors for being NEET include low socio-economic status, lack of parental support, and low educational attainment (DfE 2014, Public Health England 2014). Young people likely face similar barriers to completing treatment, as they do when accessing education and employment. They may lack a responsible adult to help organize their time and motivate them, or face physical, emotional or financial difficulties which create barriers for attending appointments. Education was the most common referral source (41%—Supplementary Table S2) with further analysis showing that fewer than five persons classed as NEET were referred by education. Whilst unsurprising, this may indicate NEET adolescents have reduced access to treatment, because they miss referral opportunities due to lack of oversight that education usually provides. It is unclear from this dataset whether alternate referral sources compensate for this.

Having a child protection plan (18.24% incomplete treatments) was also highlighted as a significant predictor of not completing treatment. A child protection plan indicates ongoing risk of significant harm due to ACEs (neglect and/or physical/sexual/emotional abuse) (NSPCC 2024). This aligns with adolescent research showing emotional neglect and/or abuse was associated with failure to complete substance use treatment (Yildiz et al. 2020). Individuals on a child protection plan commonly experience challenging family environments, parental substance/alcohol use, and a lack of parental boundaries, which pose multiple risk factors for both trauma and substance use disorders. Young people may self-medicate to cope with the experience of abuse and neglect (Becker et al. 2024), making a sustained reduction in alcohol consumption challenging.

Exiting treatment during the financial year 2020–2021 was also associated with greater odds of incomplete treatment, with the percentage of incomplete treatment rising steeply during this period before returning to its previous levels in 2021–2022 (Supplementary Table S2). The 13% increase in incomplete treatments in 2020–2021 was partly driven by a 39% decrease in treatment completions. While the impact of COVID-19 on treatment outcomes is beyond our scope, these patterns likely reflect the peak of COVID-19 related lockdowns, disrupting treatment delivery and referrals from education, the referral source with the lowest rates of incomplete treatments.

Several interrelated factors were associated with non-abstinence at treatment completion: having a mental health need, early onset alcohol use, being affected by others’ substance use, and use of illicit substances. Mental health need was reported in 39.2% of adolescents; substantially higher than population-level mental health problems in young people in England (e.g. 20.3% of 8 to 16 year-olds in 2023 (Newlove-Delgado et al. 2023). Mental health problems frequently co-occur with alcohol/substance use disorders, and their symptoms often exacerbate one another (Köck et al. 2022). This feedback loop is influenced by shared behaviours—increased impulsivity and risk taking—and disrupted neurocognitive mechanisms—reward and inhibition—which underly mental health and substance/alcohol use disorders (Groman et al. 2009, Komarnyckyj et al. 2023). These vulnerabilities may promote early experimentation with drugs and alcohol, accelerate progression into disordered use, and make it more difficult to achieve abstinence (Hamilton et al. 2019). This study further evidences the importance of integrated care models and multidisciplinary teams to improve treatment outcomes for those adolescents who experience co-occurring alcohol/substance use and mental health disorders (Mcginty and Daumit 2020, Hughes et al. 2024).

**Table 2:** Summary of Logistic Model 1 predicting incomplete treatments against complete treatments

**Predictor**	**Coefficient**	**Std. error**	**Wald *z***	***P***	**Odds ratio (OR)**	**OR lower 95% CI**	**OR upper 95% CI**
(Intercept)	−4.37	0.40	−10.87	<.001			
Age^a^	0.01	0.00	3.29	<.01	1.01^b^	1.00^b^	1.01^b^
Child protection plan	0.72	0.24	3.05	<.01	2.05	1.29	3.25
Financial year of exit 2020–2021	0.64	0.16	3.91	<.001	1.90	1.38	2.61
Monthly drinking days^c^	.27	0.05	5.45	<.001	1.31^b^	1.19^b^	1.44^b^
NEET	0.73	0.21	3.58	<.001	2.08	1.39	3.12

^a^Predictor transformed as age at treatment start squared.

^b^The odd ratios of non-binary predictors are here given for an increase of 1 in the underlying transformed variable.

^c^Predictor transformed as the square root of monthly drinking.

**Table 3:** Summary of Logistic Model 2 predicting non-abstinent completions against abstinent completions

**Predictor**	**Coefficient**	**Std. error**	**Wald *z***	***P***	**Odds ratio (OR)**	**OR lower 95% CI**	**OR upper 95% CI**
(Intercept)	−3.91	0.34	−11.66	<.001			
Age^a^	0.01	0.00	7.69	<.001	1.01^b^	1.01^b^	1.01^b^
Early onset use	0.46	0.13	3.63	<.001	1.59	1.24	2.03
Illicit substances	0.61	0.18	3.43	<.001	1.84	1.30	2.61
Mental health need	0.27	0.09	2.83	<.01	1.30	1.08	1.57
Monthly drinking days^c^	0.23	0.04	5.32	<.001	1.25^b^	1.15^b^	1.36^b^
Other’s substance use	0.35	0.12	3.00	<.01	1.41	1.13	1.77
Units per drinking day^d^	0.17	0.06	2.84	<.01	1.18^b^	1.05^b^	1.32^b^

^a^Predictor transformed as age at treatment start squared.

^b^The odd ratios of non-binary predictors are here given for an increase of 1 in the underlying transformed variable.

^c^Predictor transformed as the square root of monthly drinking days.

^d^Predictor transformed as the cube root of units per drinking day.

Being affected by others’ substance use (family or household member) suggests the young person may be exposed to increased alcohol/substance availability, inconsistent caregiving due to substance use (i.e. risk of ACEs), normalized consumption, and/or lack of parental boundaries. These environmental influences are common triggers for early onset use and may hinder the goal of abstinence during treatment. We found that 79.3% of adolescents in this cohort started drinking before age 15, whereas Government statistics show 65% of 15 years old from the general population had ever had an alcoholic drink (NHS England 2022). As early onset use and exposure to others’ substance use strongly predict later alcohol problems (Hingson and Zha 2009, Rozin and Zagonel 2012, Donoghue et al. 2017, Yuen et al. 2020, Tackaberry-Giddens et al. 2023), adolescents in these circumstances may benefit most from abstaining before adulthood.

A common theme across this study was that childhood risk factors, which may be indicators of a lack of parental boundaries/support and inconsistent caregiving, were associated with poorer treatment outcomes (e.g. NEET, child protection plan, being affected by others’ substance use). A greater emphasis may therefore be needed on strategies to enhance parental support of adolescent treatment, and address parent and child substance use in combination. We also highlight a need for trauma-informed alcohol services (Tackaberry-Giddens et al. 2023, Becker et al. 2024). For example, developing policies which ensure trauma-sensitive environments; providing specialist staff training; capturing trauma history at treatment entry (e.g. trained trauma enquiry, childhood trauma questionnaire) and subsequently referring for targeted trauma-focused interventions, e.g. Eye Movement Desensitization and Reprocessing (Walter et al. 2023, Newton-Hughes et al. 2024).

To effectively deliver these approaches and address the complex and intersecting needs of young people and their families, additional resources may be required, such as specialist support workers/therapists or funding for staff training and capacity-building (Department of Health and Social Care 2021).

### Limitations and future directions

Due to the retrospective nature of this study and its encompassing of the entire treatment population, cohort heterogeneity was relatively high compared to clinical studies. However, since we aimed to gain value from existing data, elucidating which childhood risk factors indicate increased need for additional attention or intervention, this does not undermine the results.

Given that UK Government guidelines recommend abstinence from alcohol in childhood and adolescence (consistent with policies in the USA, Australia, and much of Europe), continued drinking in this study has been regarded as a negative outcome of treatment. However, the lack of clarity around treatment goals (abstinence intention vs. harm reduction) made it difficult to determine whether non-abstinent completions reflect negative outcomes from the individual’s/treatment provider’s perspective. Absence of long-term follow-up information prevented investigation of the long-term effects of treatment.

These limitations could be addressed through future collaboration with treatment providers, permitting inclusion of additional data in NDTMS and complementary qualitative evidence on how risk factors are currently addressed and experienced. More advanced methods might disentangle the effect of existing multi-agency interventions already being delivered alongside alcohol treatment (e.g. peer/family/housing support).

## Conclusion

Our findings indicate that adolescents with an increased risk of long-term alcohol use problems, due to higher use at treatment start, have worse treatment outcomes in England. Indicators of low socioeconomic status (NEET) and ACEs (being placed on a child protection plan) were associated with reductions in completed treatments. Exposure to environments potentially encouraging the formation of maladaptive norms (others’ substance use, illicit substance use) pose barriers for abstinent treatment completion. These groups are highlighted as targets for tailored interventions which consider psychological and environmental challenges adolescents may be experiencing.

## Supplementary Material

agaf081_adolescent_outcomes_supporting_information_v2

## Data Availability

The authors are not permitted to share research data due to government data publishing restrictions.
