# Supplementary material for: Associations between childhood risk factors and alcohol treatment outcomes in adolescence
Source: Alcohol Alcohol. 2026 Jan 15;61(2):agaf081. doi: 10.1093/alcalc/agaf081 (PMC12803731; doi:10.1093/alcalc/agaf081)
Supplement: agaf081_adolescent_outcomes_supporting_information_v2 [file agaf081_adolescent_outcomes_supporting_information_v2.docx]

**Associations between childhood risk factors and alcohol treatment outcomes in adolescence**

Mica Komarnyckyj^1^*^, Dylan Mangan^2^^, Karen P. Hayhurst^2^, Stephen J Kaar^3-5^, Stefan Jahr^6^, Andrew Jones^2^

^1^ Biomedical Research Centre, Division of Psychology & Mental Health, University of Manchester, Oxford Rd, Manchester, M13 9PL

^2^ National Drug Evidence Centre, Division of Population Health, Health Service Research and Primary Care, University of Manchester, Manchester M13 9PL, UK

^3^ Division of Psychology & Mental Health, University of Manchester, Oxford Rd, Manchester, M13 9PL

^4^ Greater Manchester Mental Health NHS Foundation Trust, Addictions Services, Manchester, M25 3BL

^5^ Addictions Lead, Royal College of Psychiatrists Child and Adolescent Mental Health Faculty Executive Committee

^6^ Department of Health and Social Care, Office for Health Improvement and Disparities (OHID), London, UK

^^^ Authors equally contributed to this work

* Corresponding author: Mica Komarnyckyj (telephone: 0161 306 6000, address: G.803 Stopford Building, University of Manchester, Oxford Road, Manchester, M13 9PT, email: [Mica.Komarnyckyj@manchester.ac.uk](mailto:Mica.Komarnyckyj@manchester.ac.uk))

**Graphical Abstract**


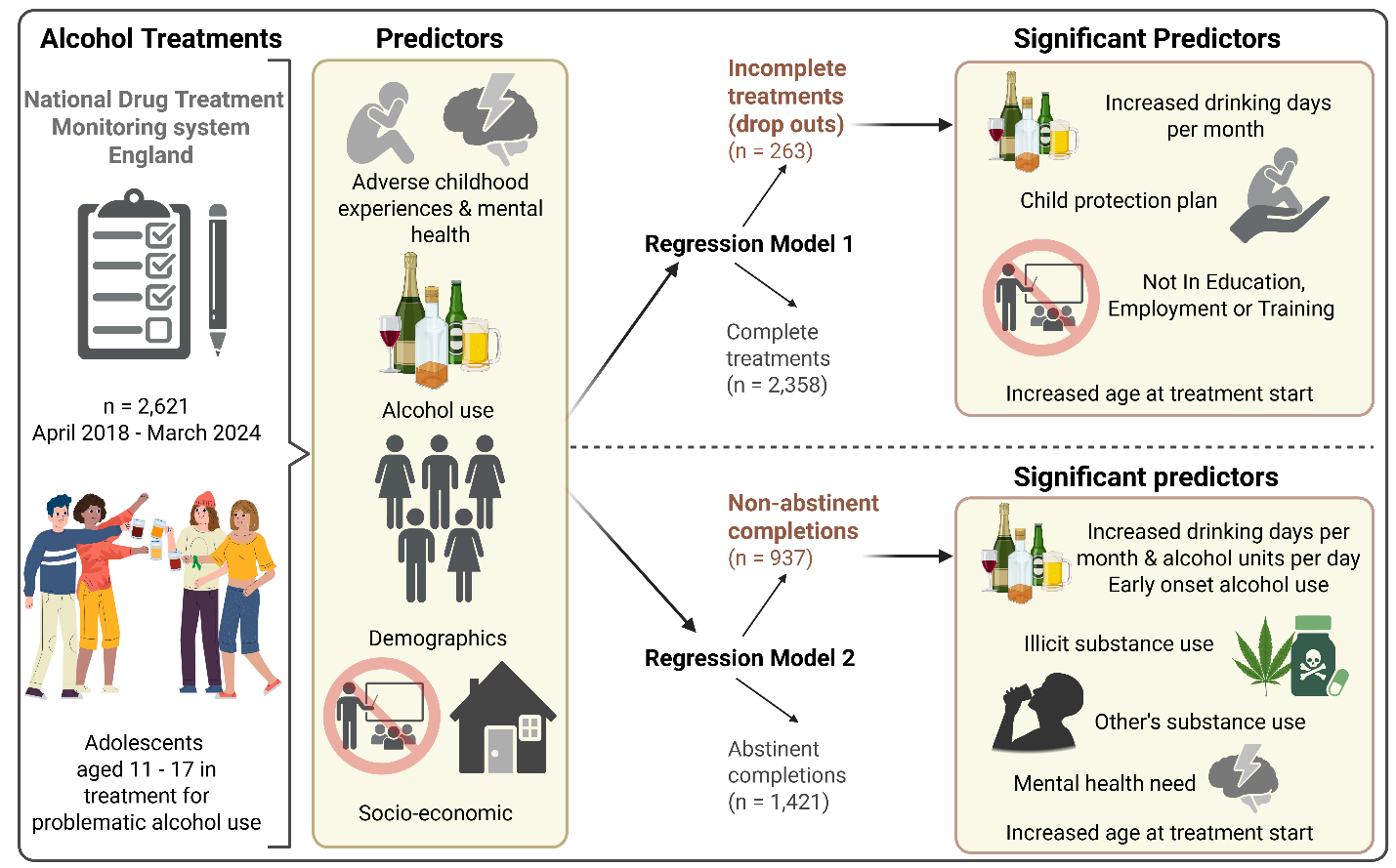


Created in BioRender. Komarnyckyj, M. (2025) https://BioRender.com/uhztl3q

# Supporting methods

## **Treatment Journey**

All agencies in receipt of public funding for the treatment of drug and alcohol abuse problems are required to submit data each month on all the treatments they deliver to individuals to the Office for Health Improvement and Disparities (OHID), in the Department of Health and Social Care (DHSC). Data is captured at treatment start, treatment end, and at regular intervals during a “treatment episode”, which is typically a single treatment type (i.e. psychosocial, pharmaceutical), at a given treatment provider. A single psychosocial episode may consist of several separate psychosocial interventions, for example combining cognitive behavioural therapy (CBT) with motivational interventions, depending on what the treatment provider has determined to be appropriate.

“A treatment journey consists of one or more episodes of structured treatment, at one or more providers, where there has been less than 21 days break between treatment episodes. A treatment journey ends once a client has been exited entirely from structured drug/alcohol treatment once all structured interventions and the episode have been closed. A client may be discharged from one provider but if they continue structured treatment (within 21 days of discharge) at another provider, their NDTMS treatment journey is continued.”

“If a client is discharged from treatment with a discharge reason of ‘treatment completed’ this indicates that the client has no further structured treatment need. Therefore, this should only be used at the end of a client’s treatment journey when they have completed structured treatment at all providers.” (NHS England; Office for Health & Improvement Disparities, 2023).

## **Treatment Outcome Profile (TOP)**

This is a form submitted regularly during an individual’s treatment(s), the data included here specifically relates to the predictors “Monthly Drinking Days” and “Units per Occasion”. TOP data is kept in a separate database to most NDTMS indicators and requires matching in. In some cases, this means no TOP is identified to match with an individual’s treatment.

## **Data Slection – Excluded Data**

**Supporting Table 1: The number of adolescents’ treatment journeys removed from the dataset.**

| Full cohort extracted before exclusions |  | 3129 |
| --- | --- | --- |
| **Exclusion criteria** | **Excluded** | **Remaining** |
| 1) Treatment journey over 24 months` | 8 | 3121 |
| 2) Not a first journey (the individual had multiple journeys) | 242 | 2879 |
| 3) No treatment outcomes profile record | 56 | 2823 |
| 4) No record of 'Monthly Drinking Days' | 197 | 2626 |
| 5) No record of 'Units per Occasion' | 5 | 2621 |

## **Detailed description of Risk Factors & Alcohol use variables**

Below are the questions and answers used to define the childhood risk factors used in this study. These are asked of an individual by providers as they begin each treatment episode and reported to the NDTMS. The majority of these are calculated using data provided by the client at the beginning of their treatment. The quoted information has been extracted from the NDTMS, Reference Data (Core Dataset Q, OHID, 2023) (NHS England; Office for Health & Improvement Disparities, 2023) a significant amount of additional guidance and documentation is provided by OHID to ensure consistency across treatment agencies.

**Psychological factors**

- Mental Health Need – “Does the client have a mental health treatment need?” [Answer: YES]
- Self-Harm – “Has the Young Person (YP) self-harmed prior to treatment start?” [Answer: YES]

**Adverse childhood experiences**

- Witnessing Domestic Abuse – “Has the client ever been the victim of domestic abuse?” [Answer: “Yes – currently (last 28 days)” OR “Yes – previously” OR “Yes – currently and previously”]. The NDTMS dataset recognises children as victims of domestic abuse if they “see, hear or otherwise experience the effects of abuse” and are related to either the abuser or abused. The term “Abusive” can refer to: physical or sexual abuse; violent or threatening behaviour; controlling or coercive behaviour and gaslighting; economic abuse; psychological abuse; female genital mutilation (FGM); ‘honour-based' violence and more.
- Other’s Substance Use – “Does the young person feel affected by substance misuse in their close family/members of their household at treatment start?” [Answer: “Yes”]
- Sexual Exploitation – “Has the YP been affected by child criminal exploitation at treatment start?” [Answer: Yes]

**Care Status - Indicator of prior adverse childhood experiences**

- Child In Need – “What was the care status of the YP at treatment start?” [Answer: Child In Need]. This means a social worker has assessed the child and family, determining the child needs help or protection due to risks to their health or development.
- Looked After Child – “What was the care status of the YP at treatment start?” [Answer: Looked after child]. This means the child has been in local authority care for over 24 hours (e.g., foster care, children’s home, secure accommodation, or with prospective adopters), following statutory intervention and care proceedings under the Children Act 1989, which may occur due to parental inability to look after the child, child welfare concerns, or criminal charges.
- Child Protection Plan – “Was the YP subject to a Child Protection Plan (CPP) at treatment start? [Answer: Is currently subject to Child Protection Plan (CPP)]. The child is currently deemed to be at risk of significant harm, as determined by a child protection conference. A multi-agency Child Protection Plan is in place, and a social worker has conducted Section 47 enquiries under the Children Act 1989.

**Behavioural factors**

- Early Onset Use – Age first used Alcohol [Answer < 15]
- Anti-Social Behaviour – “Has YP been involved in antisocial behaviour or committed a criminal act on more than 1 occasion in the past 6 months?” [Answer: Yes]
- Illicit Substances - Used illicit substances which they are not in treatment for, in the 28 days prior to treatment. [This variable is calculated where a client reports having used an illicit substance in the 28 days prior to treatment. In this study, it implies the individual is not receiving treatment specifically for any of these substances, as the population has been restricted to those receiving treatment for alcohol only.]

**Social and economic factors**

- NEET – “YP’s education/employment/training status at treatment start” [Answer: Not in employment or education or training (NEET)]
- Housing Problem - Accommodation Need [Answer = 28 “Client living independently in unsettled accommodation” or Answer = 29 “Client living independently with no fixed abode”]
- Pregnant Or Parent –
  - Is pregnant [Answer = Yes and Sex = Female], or.
  - Is a parent. [ “If client has parental responsibility, do any of these children live with the client?” Answer = 11 “All the children live with the client” or Answer = 12 “Some of the children live with the client” or Answer = 13 “None of the children live with the client”]

**Alcohol Use in the 28 days prior to treatment entry**

- The number of days on which the client reports having consumed alcohol [0-28].
- The mean alcohol units on each of these drinking days, i.e. on average how many units have they consumed on each occasion [0-200].

## **Excluded Risk factors**

Several routinely collected childhood vulnerabilities were not included in the analysis for specific reasons:

- High risk alcohol – This binary variable was excluded, in favour of including the constituent variables “The number of days they had consumed alcohol.” and “The mean alcohol units on each of these drinking days”. This permitted elucidation of their different effects.
- Homelessness, unsafe sex, involved in gangs, criminal exploitation – data used to calculate these variables only began to be collected part way through the time period being analysed, these therefore had to be excluded in favour of not reducing the size of the sample.
- Injecting, Opiate/crack use – These were excluded due to extremely low numbers in the sample.

## **Mapping outcomes to NDTMS discharge codes**

Journey exits are reported to National Drug Treatment Monitoring System (NDTMS) using specific discharge reason codes and grouped as per published information. A “successful completion” (referred to as a “completion” in this study), is usually reported as either “completed – alcohol/drug-free” or “Treatment completed – occasional user (not opiates or crack)”. For the purpose of this study, the usually reported category has been split into these two constituents. “Incomplete treatments” are those which are not reported as successfully completed and had not been transferred to a new treatment provider without being “picked up” by the new treatment provider for over 21 days (NHS England; Office for Health & Improvement Disparities, 2023).

## **Data processing Software**

Data processing was conducted in SPSS 29 (IMB Corp. Released 2023. IBM SPSS Statistics for Windows, Version 29.0.2.0 Armonk, NY: IBM Cor*p*) analysis in R (R Core Team, 2024), using R studio ("Posit team," 2024) and packages: companion to applied regression (Fox & Weisberg, 2019), readr (Wickham et al., 2021), dplyr (Wickham et al., 2024), ggplot2 (Wickham, 2016), scales (Wickham et al., 2023), car, reshape (Wickham, 2007), data.table, glmtoolbox (Vanegas et al., 2024) and DHARMa (Hartig, 2022).

# ****Supporting Results****

## **Outliers & goodness of fit**

Both Model 1 and Model 2 were assessed using the DHARMa package in R. The results are presented in Supporting Figures 1 and 2 (Model 1) and Supporting Figures 3 and 4 (Model 2)

Supporting Figure 1: QQ plot of residuals to uniform distribution for Model 1

**
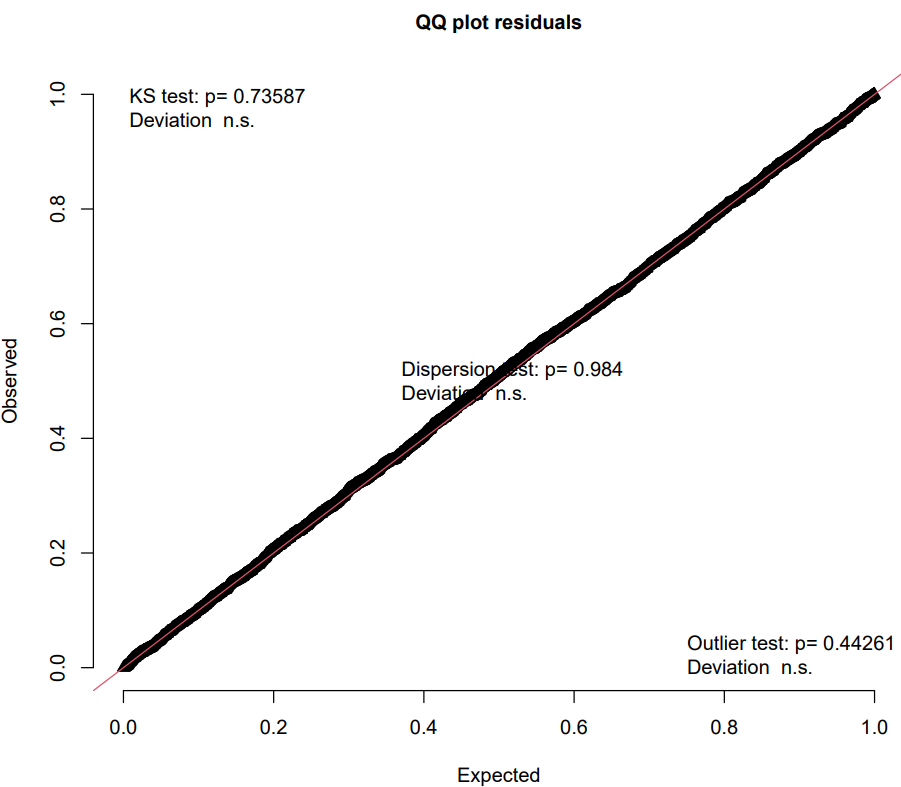
**

Supporting Figure 2: Residuals to Prediction plot showing quantiles for Model 1 (*p* = 0.74)

**
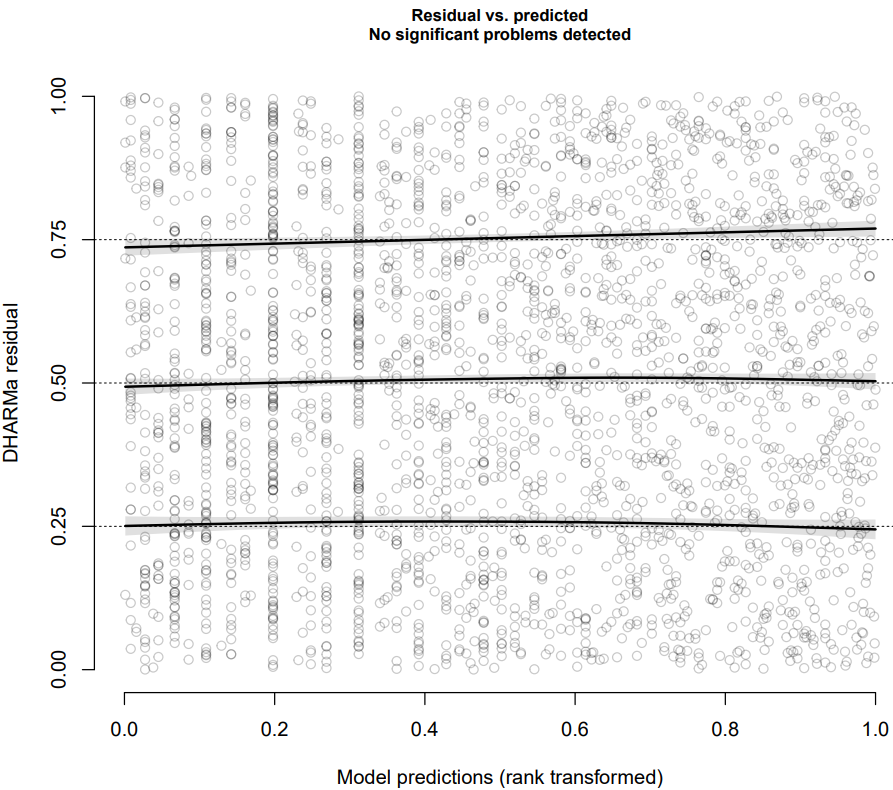
**

Supporting Figure 3: QQ plot of residuals to uniform distribution for Model 2

**
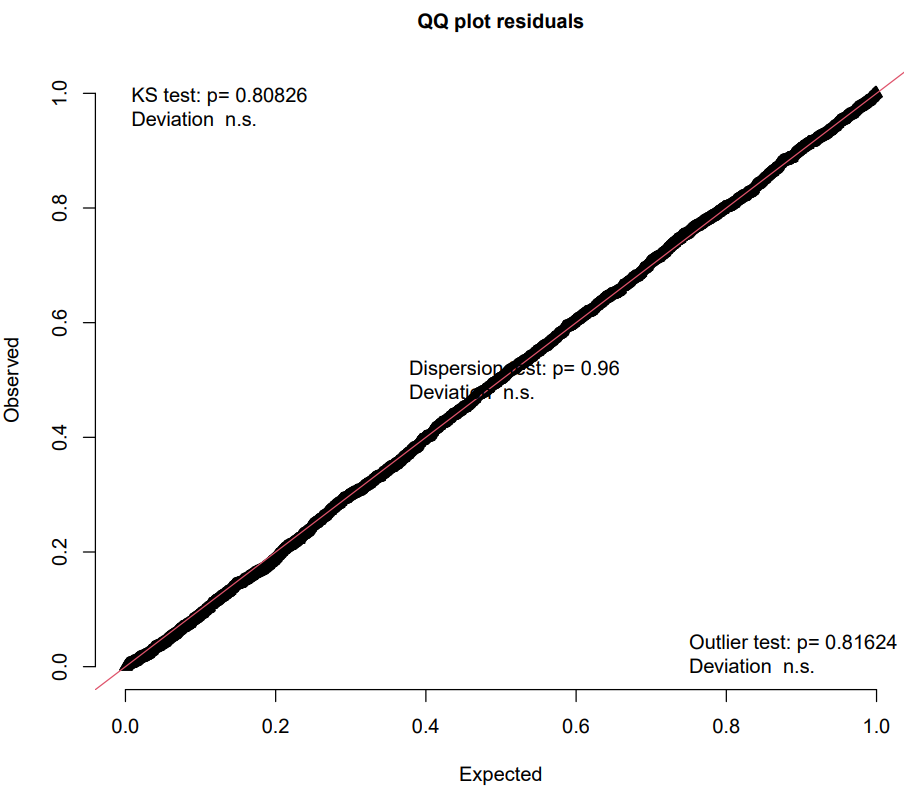
**

Supporting Figure 4: Residuals to Prediction plot showing quantiles for Model 2 (*p* = 0.85)

**
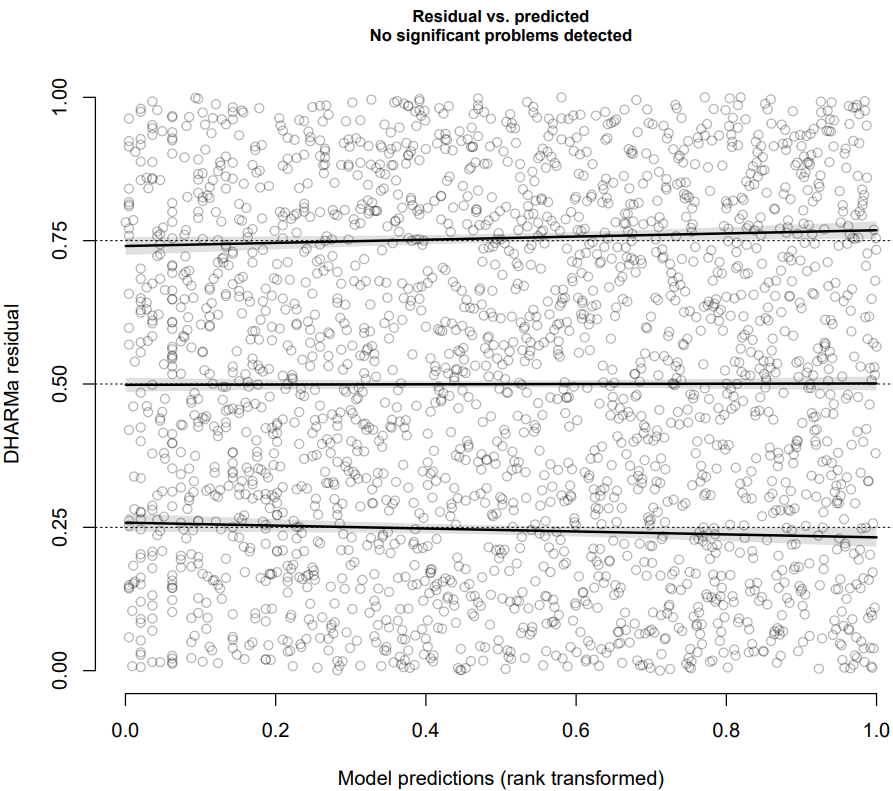
**

## **Odds Ratio Interpretation**

Odds ratios of **binary predictors** represent a Y/N of an underlying variable, the majority of those in the models. Interpretation is straight forward, for example an odds ratio of 2.05 for the predictor of “child protection plan”, means the odds of a client exiting treatment before completion are 2.05 times those of an individual where other predictors are held constant, i.e. an individual in identical circumstances except for the child protection plan.

Interpreting odds ratios becomes more complicated for **ordinal interval and continuous predictors** (e.g. age, drinking days and units per drinking occasion in the models presented here). In these cases, an odds ratio of 1.08 represents an increase in the odds of not completing treatment for *an increase of one* in the underlying variable, where other predictors are held constant. In this situation, an increase of one in the underlying predictor may be smaller than the real-world differences one might expect to see, i.e. an increase of 10 in the underlying variable could be more meaningful than an increase of 1 in interpreting results. Increases of size $d$ > 1 are calculated using the formula:

$${OR}_{d}= e^{d.ln({OR}_{1})}$$

Where: ${OR}_{d}$ is the odds ratio for an increase of $d$ in the underlying predictor for which the odds ratio of interest is being calculated, ${OR}_{1}$ is the odds ratio for an increase of one in that predictor. Using this formula, an increase of ten, regardless of whether this is an increase from one to eleven or five to fifteen, in an underlying predictor with an odds ratio of 1.08 for an increase of 1 in the underlying variable, would be calculated as:

$${OR}_{10}= e^{10.\ln\left( 1.08 \right)}=2.16$$

This demonstrates how odds ratios for ordinal/interval/continuous predictors which are small prima facie, may in reality have a much larger effect.

The situation is further complicated for **ordinal/interval/continuous predictors** which have been transformed from their real-world distributions, here to address skew. This change in the distribution means an odds ratio for an increase from one to eleven in the real world variables is no longer equivalent to an increase from five to fifteen. In order to provide meaningful odds ratios for real world increases in underlying variables using the transformed versions of these variables, the above formula has been adapted to:

$${OR}_{b-a}= e^{(f\left( b \right)-f\left( a \right)).ln({OR}_{1})}$$

Where ${OR}_{b-a}$ is the odds ratio of an increase from a to b; $f$ is the function used to transform the real-world variable to the predictor variable used in the model; ${OR}_{1}$ is the odds ratio for an increase of one in that predictor (i.e. the transformed real-world variable).

For our purpose, the variable “Monthly days drinking”, was transformed to $\sqrt[2]{Monthly Drinking Days}$, and included as a predictor. For example, we can calculate, using the results from our study for predicting incomplete treatments, the odds ratios representing increases from four to eight days per month (i.e. moving from one day a week to two) and from eight to twelve days as:

$${OR}_{8-4}= e^{(\surd8-\surd4).ln(1.31)}=e^{0.83.ln(1.31)} 1.25$$

$${OR}_{12-8}= e^{(\surd12-\surd8).ln(1.31)}=e^{0.64.ln(1.31)} 1.19$$

This demonstrates how the values in Table 3 (main manuscript) may seem smaller than expected for age, which was transformed to ${Age}^{2}$ and included as a predictor. It also shows how an odds ratio for an increase of the same amount (here four) in the real-world predictor “monthly drinking days”, may differ depending on the value from which the increase of four is being calculated from.

## **Supporting Tables for the Main Results**

Supporting Table 2: Descriptive statistics for childhood risks across the whole sample of exited treatments and split by incomplete vs complete treatments.

|  | **Whole sample prevalence** | | **Incomplete Treatment Group** | | **Completed Treatment Group** | |
| --- | --- | --- | --- | --- | --- | --- |
| **Variable** | **n** | **% of sample** | **n** | **% of sample** | **n** | **% of sample** |
| All Individuals | 2621 | 100 | 263 | 10.03 | 2358 | 89.97 |
| Female | 1640 | 62.57 | 172 | 10.49 | 1468 | 89.51 |
| Referral: Education | 1083 | 41.32 | 58 | 5.36 | 1025 | 94.64 |
| Referral: Criminal Justice | 285 | 10.87 | 35 | 12.28 | 250 | 87.72 |
| Referral: Self | 117 | 4.46 | 17 | 14.53 | 100 | 85.47 |
| Referral: Relative/Friend | 74 | 2.82 | 8 | 10.81 | 66 | 89.19 |
| Referral: Social Care | 480 | 18.31 | 66 | 13.75 | 414 | 86.25 |
| Referral: Substance Misuse Service | 41 | 1.56 | 9 | 21.95 | 32 | 78.05 |
| Referral: Health Services | 468 | 17.86 | 61 | 13.03 | 407 | 86.97 |
| Referral: Other | 73 | 2.79 | 9 | 12.33 | 64 | 87.67 |
| Behavioural Disability | 43 | 1.64 | 5 | 11.63 | 38 | 88.37 |
| Financial Year of Exit 18/19 | 485 | 18.5 | 39 | 8.04 | 446 | 91.96 |
| Financial Year of Exit 19/20 | 544 | 20.76 | 55 | 10.11 | 489 | 89.89 |
| Financial Year of Exit 20/21 | 362 | 13.81 | 62 | 17.13 | 300 | 82.87 |
| Financial Year of Exit 21/22 | 558 | 21.29 | 54 | 9.68 | 504 | 90.32 |
| Financial Year of Exit 22/23 | 672 | 25.64 | 53 | 7.89 | 619 | 92.11 |

Supporting Table 3: Initial pre-step Logistic Model 1, predicting incomplete compared to complete treatments. Odds ratios > 1 implies increased chance of an incomplete treatment.

| **Predictor** | **Estimate** | **Std. Error** | **Wald z** | ***p*** | **Generalised Variance**  **Inflation Factor** | **R^2^ coefficient of determination with other predictors** | **Odds Ratio (OR)** | **OR Lower 95% CI** | **OR Upper 95% CI** |
| --- | --- | --- | --- | --- | --- | --- | --- | --- | --- |
| (Intercept) | -4.39 | 0.55 | -8.05 | <0.001 |  |  |  |  |  |
| Age^#^ | 0.00 | 0.00 | 2.01 | 0.04 | 1.70 | 0.41 | 1.00 ^α^ | 1.00 ^α^ | 1.01 ^α^ |
| Anti-Social Behaviour | 0.04 | 0.20 | 0.20 | 0.84 | 1.37 | 0.27 | 1.04 | 0.71 | 1.53 |
| Behavioural Disability | 0.19 | 0.52 | 0.38 | 0.71 | 1.08 | 0.07 | 1.22 | 0.44 | 3.34 |
| Child in Need | 0.01 | 0.25 | 0.04 | 0.97 | 1.16 | 0.14 | 1.01 | 0.62 | 1.65 |
| Child Protection Plan | 0.64 | 0.25 | 2.56 | 0.01 | 1.14 | 0.12 | 1.89 | 1.16 | 3.08 |
| Domestic Abuse | 0.01 | 0.18 | 0.05 | 0.96 | 1.20 | 0.17 | 1.01 | 0.71 | 1.44 |
| Early Onset Use | -0.17 | 0.17 | -0.99 | 0.32 | 1.27 | 0.21 | 0.84 | 0.60 | 1.18 |
| Female | 0.21 | 0.15 | 1.36 | 0.17 | 1.16 | 0.14 | 1.23 | 0.91 | 1.66 |
| Financial Year of Exit 19/20 | 0.14 | 0.23 | 0.61 | 0.54 | 1.89 | 0.47 | 1.15 | 0.74 | 1.79 |
| Financial Year of Exit 20/21 | 0.54 | 0.23 | 2.36 | 0.02 | 1.96 | 0.49 | 1.72 | 1.10 | 2.70 |
| Financial Year of Exit 21/22 | 0.00 | 0.23 | -0.01 | 0.99 | 1.94 | 0.48 | 1.00 | 0.63 | 1.57 |
| Financial Year of Exit 22/23 | -0.20 | 0.24 | -0.87 | 0.39 | 2.03 | 0.51 | 0.82 | 0.51 | 1.29 |
| Housing Problem | 0.40 | 0.26 | 1.53 | 0.13 | 1.08 | 0.07 | 1.49 | 0.89 | 2.50 |
| Illicit Substances | -0.29 | 0.26 | -1.13 | 0.26 | 1.06 | 0.06 | 0.75 | 0.45 | 1.24 |
| Looked After Child | 0.44 | 0.23 | 1.96 | 0.05 | 1.24 | 0.19 | 1.55 | 1.00 | 2.42 |
| Mental Health Need | -0.01 | 0.16 | -0.04 | 0.97 | 1.38 | 0.28 | 0.99 | 0.73 | 1.36 |
| Monthly Drinking Days* | 0.26 | 0.06 | 4.54 | <0.001 | 1.60 | 0.38 | 1.30 ^α^ | 1.16 ^α^ | 1.45 ^α^ |
| NEET | 0.61 | 0.22 | 2.83 | <0.01 | 1.23 | 0.19 | 1.85 | 1.21 | 2.83 |
| Other’s Substance Use | 0.11 | 0.17 | 0.66 | 0.51 | 1.16 | 0.14 | 1.12 | 0.80 | 1.56 |
| Pregnant or Parent | -0.03 | 0.37 | -0.09 | 0.93 | 1.03 | 0.03 | 0.97 | 0.47 | 2.00 |
| Referral: Criminal Justice | 0.40 | 0.27 | 1.45 | 0.15 | 1.88 | 0.47 | 1.49 | 0.87 | 2.55 |
| Referral: Health Services | 0.59 | 0.21 | 2.83 | <0.01 | 1.66 | 0.40 | 1.80 | 1.20 | 2.70 |
| Referral: Other | 0.18 | 0.41 | 0.44 | 0.66 | 1.20 | 0.17 | 1.20 | 0.54 | 2.67 |
| Referral: Relative Friend | 0.25 | 0.42 | 0.59 | 0.56 | 1.13 | 0.12 | 1.28 | 0.56 | 2.93 |
| Referral: Self | 0.44 | 0.32 | 1.39 | 0.17 | 1.29 | 0.22 | 1.55 | 0.83 | 2.90 |
| Referral: Social Care | 0.57 | 0.21 | 2.69 | 0.01 | 1.78 | 0.44 | 1.76 | 1.17 | 2.66 |
| Referral: Substance Misuse Service | 1.11 | 0.43 | 2.59 | 0.01 | 1.12 | 0.11 | 3.03 | 1.31 | 7.02 |
| Self-Harm | 0.10 | 0.17 | 0.58 | 0.56 | 1.30 | 0.23 | 1.10 | 0.80 | 1.52 |
| Sex Exploitation | -0.77 | 0.38 | -2.02 | 0.04 | 1.09 | 0.08 | 0.46 | 0.22 | 0.98 |
| Units per drinking day^ | 0.02 | 0.09 | 0.26 | 0.79 | 1.40 | 0.29 | 1.02 ^α^ | 0.86 ^α^ | 1.22 ^α^ |

^#^Predictor transformed from (Age at treatment start)^2^

*Predictor transformed from $\sqrt[2]{Monthly Drinking Days}$

^ Predictor transformed from $\sqrt[3]{Units per drinking day}$

^α^ The Odd ratios of non-binary predictors are here given for an increase of 1 in the underlying transformed predictor

Supporting Table 4: Back transformed odd ratios for significant interval predictors from Model 1:incomplete vs complete treatments. Shows relevant odds ratios for real world increases in the underlying variables (age and drinking days) providing a better understanding of their effect sizes. Lower Quartile (LQ); Upper Quartile (UQ); Confidence Interval (CI).

| **Significant Predictor Model 1: Age** | | | |
| --- | --- | --- | --- |
| Real world comparison | Odds Ratio | Lower 95% CI | Upper 95% CI |
| 15-year-old relative to 14-year-old (median to LQ) | 1.19 | 1.07 | 1.31 |
| 16-year-old relative to 15-year-old (UQ to median) | 1.20 | 1.08 | 1.34 |
| 16-year-old relative to 14-year-old (UQ to LQ) | 1.42 | 1.15 | 1.75 |
| **Significant Predictor Model 1: Monthly Drinking Days** | | | |
| Real world comparison | Odds Ratio | Lower 95% CI | Upper 95% CI |
| 2 drinking days per month relative to 1 (median to LQ) | 1.12 | 1.07 | 1.16 |
| 8 drinking days per month relative to 2 (UQ to median) | 1.46 | 1.27 | 1.67 |
| 8 drinking days per month relative to 1 (UQ to LQ) | 1.63 | 1.37 | 1.94 |

Supporting Table 5: Descriptive statistics for childhood risks across the subsample of completed treatments and split by abstinent completions vs non-abstinent completions.

|  | **Subsample prevalence** | | **Non-abstinent Completions** | | **Abstinent completions** | |
| --- | --- | --- | --- | --- | --- | --- |
| **Variable** | **n** | **% of sample** | **n** | **% of sample** | **n** | **% of sample** |
| All Individuals | 2358 | 100 | 937 | 39.74 | 1421 | 60.26 |
| Female | 1468 | 62.26 | 571 | 38.9 | 897 | 61.1 |
| Referral: Education | 1025 | 43.47 | 324 | 31.61 | 701 | 68.39 |
| Referral: Criminal Justice | 250 | 10.6 | 119 | 47.6 | 131 | 52.4 |
| Referral: Self | 100 | 4.24 | 60 | 60 | 40 | 40 |
| Referral: Relative/Friend | 66 | 2.8 | 26 | 39.39 | 40 | 60.61 |
| Referral: Social Care | 414 | 17.56 | 177 | 42.75 | 237 | 57.25 |
| Referral: Substance Misuse Service | 32 | 1.36 | 16 | 50 | 16 | 50 |
| Referral: Health Services | 407 | 17.26 | 183 | 44.96 | 224 | 55.04 |
| Referral: Other | 64 | 2.71 | 32 | 50 | 32 | 50 |
| Behavioural Disability | 38 | 1.61 | 19 | 50 | 19 | 50 |
| Early Onset Use | 1887 | 80.03 | 754 | 39.96 | 1133 | 60.04 |
| Illicit Substances | 163 | 6.91 | 104 | 63.8 | 59 | 36.2 |
| Anti-Social Behaviour | 371 | 15.73 | 186 | 50.13 | 185 | 49.87 |
| Mental Health Need | 896 | 38 | 434 | 48.44 | 462 | 51.56 |
| Other's Substance Use | 438 | 18.58 | 215 | 49.09 | 223 | 50.91 |
| Domestic Abuse | 360 | 15.27 | 169 | 46.94 | 191 | 53.06 |
| NEET | 126 | 5.34 | 69 | 54.76 | 57 | 45.24 |
| Self-Harm | 600 | 25.45 | 277 | 46.17 | 323 | 53.83 |
| Looked After Child | 170 | 7.21 | 79 | 46.47 | 91 | 53.53 |
| Child in Need | 183 | 7.76 | 71 | 38.8 | 112 | 61.2 |
| Child Protection Plan | 121 | 5.13 | 43 | 35.54 | 78 | 64.46 |
| Sex Exploitation | 92 | 3.9 | 44 | 47.83 | 48 | 52.17 |
| Pregnant or Parent | 66 | 2.8 | 27 | 40.91 | 39 | 59.09 |
| Housing Problem | 116 | 4.92 | 42 | 36.21 | 74 | 63.79 |
| Financial Year of Exit 18/19 | 446 | 18.91 | 160 | 35.87 | 286 | 64.13 |
| Financial Year of Exit 19/20 | 489 | 20.74 | 186 | 38.04 | 303 | 61.96 |
| Financial Year of Exit 20/21 | 300 | 12.72 | 116 | 38.67 | 184 | 61.33 |
| Financial Year of Exit 21/22 | 504 | 21.37 | 204 | 40.48 | 300 | 59.52 |
| Financial Year of Exit 22/23 | 619 | 26.25 | 271 | 43.78 | 348 | 56.22 |

Supporting Table 6: Initial pre-step Logistic Model 2, predicting non-abstinent compared to abstinent completion. Odds ratios >1 gives increased chance of non-abstinent completion.

| **Predictor** | **Estimate** | **Std. Error** | **Wald z** | **p** | **Generalised Variance**  **Inflation Factor** | **R^2^ coefficient of determination with other predictors** | **Odds Ratio (OR)** | **OR Lower 95% CI** | **OR Upper 95% CI** |
| --- | --- | --- | --- | --- | --- | --- | --- | --- | --- |
| (Intercept) | -3.82 | 0.36 | -10.52 | <0.001 |  |  |  |  |  |
| Age# | 0.01 | 0.00 | 6.60 | <0.001 | 1.59 | 0.37 | 1.01 α | 1.01 α | 1.01 α |
| Anti-Social Behaviour | 0.26 | 0.14 | 1.88 | 0.06 | 1.33 | 0.25 | 1.30 | 0.99 | 1.71 |
| Behavioural Disability | -0.25 | 0.36 | -0.67 | 0.50 | 1.07 | 0.07 | 0.78 | 0.38 | 1.60 |
| Child in Need | -0.18 | 0.18 | -0.96 | 0.33 | 1.15 | 0.13 | 0.84 | 0.59 | 1.20 |
| Child Protection Plan | -0.25 | 0.22 | -1.15 | 0.25 | 1.11 | 0.10 | 0.78 | 0.50 | 1.19 |
| Domestic Abuse | 0.12 | 0.14 | 0.91 | 0.36 | 1.18 | 0.15 | 1.13 | 0.87 | 1.47 |
| Early Onset Use | 0.46 | 0.13 | 3.59 | <0.001 | 1.32 | 0.24 | 1.59 | 1.23 | 2.04 |
| Female | -0.03 | 0.10 | -0.30 | 0.76 | 1.13 | 0.12 | 0.97 | 0.80 | 1.18 |
| Financial Year of Exit 19/20 | -0.10 | 0.15 | -0.71 | 0.48 | 1.71 | 0.42 | 0.90 | 0.68 | 1.20 |
| Financial Year of Exit 20/21 | -0.24 | 0.17 | -1.42 | 0.16 | 1.55 | 0.35 | 0.79 | 0.56 | 1.10 |
| Financial Year of Exit 21/22 | 0.00 | 0.15 | 0.01 | 0.99 | 1.77 | 0.44 | 1.00 | 0.75 | 1.33 |
| Financial Year of Exit 22/23 | 0.16 | 0.14 | 1.15 | 0.25 | 1.96 | 0.49 | 1.18 | 0.89 | 1.56 |
| Housing Problem | -0.52 | 0.22 | -2.38 | 0.02 | 1.06 | 0.06 | 0.59 | 0.38 | 0.91 |
| Illicit Substances | 0.58 | 0.18 | 3.20 | <0.01 | 1.04 | 0.04 | 1.79 | 1.25 | 2.55 |
| Looked After Child | -0.08 | 0.19 | -0.41 | 0.68 | 1.16 | 0.14 | 0.93 | 0.64 | 1.34 |
| Mental Health Need | 0.25 | 0.11 | 2.38 | 0.02 | 1.35 | 0.26 | 1.29 | 1.05 | 1.59 |
| Monthly Drinking Days* | 0.23 | 0.04 | 5.26 | <0.001 | 1.49 | 0.33 | 1.26 α | 1.15 α | 1.37 α |
| NEET | -0.08 | 0.21 | -0.36 | 0.72 | 1.15 | 0.13 | 0.92 | 0.61 | 1.41 |
| Other’s Substance Use | 0.37 | 0.12 | 3.01 | <0.01 | 1.14 | 0.12 | 1.45 | 1.14 | 1.84 |
| Pregnant or Parent | -0.21 | 0.27 | -0.77 | 0.44 | 1.02 | 0.02 | 0.81 | 0.47 | 1.39 |
| Referral: Criminal Justice | 0.28 | 0.18 | 1.57 | 0.12 | 1.58 | 0.37 | 1.33 | 0.93 | 1.89 |
| Referral: Health Services | 0.26 | 0.13 | 1.94 | 0.05 | 1.30 | 0.23 | 1.30 | 1.00 | 1.68 |
| Referral: Other | 0.24 | 0.29 | 0.84 | 0.40 | 1.11 | 0.10 | 1.27 | 0.72 | 2.23 |
| Referral: Relative Friend | 0.04 | 0.28 | 0.14 | 0.89 | 1.06 | 0.06 | 1.04 | 0.60 | 1.79 |
| Referral: Self | 0.57 | 0.24 | 2.39 | 0.02 | 1.12 | 0.11 | 1.77 | 1.11 | 2.82 |
| Referral: Social Care | 0.20 | 0.14 | 1.45 | 0.15 | 1.38 | 0.28 | 1.22 | 0.93 | 1.60 |
| Referral: Substance Misuse Service | 0.43 | 0.40 | 1.09 | 0.28 | 1.04 | 0.04 | 1.54 | 0.71 | 3.36 |
| Self-Harm | -0.07 | 0.12 | -0.61 | 0.54 | 1.33 | 0.25 | 0.93 | 0.74 | 1.17 |
| Sex Exploitation | 0.08 | 0.24 | 0.32 | 0.75 | 1.12 | 0.11 | 1.08 | 0.67 | 1.74 |
| Units per drinking day^ | 0.16 | 0.06 | 2.71 | 0.01 | 1.37 | 0.27 | 1.17 α | 1.05 α | 1.32 α |

^#^Predictor transformed from (Age at treatment start)^2^

*Predictor transformed from $\sqrt[2]{Monthly Drinking Days}$

^ Predictor transformed from $\sqrt[3]{Units per drinking day}$

^α^ The Odd ratios of non-binary predictors are here given for an increase of 1 in the underlying transformed predictor

**Supporting Table 7: Back transformed odd ratios for significant interval predictors from Model 2: non-abstinent vs abstinent completions.** Shows relevant odds ratios for real world increases in the underlying variables (age, drinking days and units per occasion) providing a better understanding of their effect sizes. Lower Quartile (LQ); Upper Quartile (UQ); Confidence Interval (CI). Lower Quartile (LQ); Upper Quartile (UQ); Confidence Interval (CI).

| **Significant Predictor Model 2: Age** | | | |
| --- | --- | --- | --- |
| Real world comparison | Odds Ratio | Lower 95% CI | Upper 95% CI |
| 15-year-old relative to 14-year-old (median to LQ) | 1.33 | 1.24 | 1.43 |
| 16-year-old relative to 15-year-old (UQ to median) | 1.35 | 1.25 | 1.46 |
| 16-year-old relative to 14-year-old (UQ to LQ) | 1.80 | 1.55 | 2.09 |
| **Significant Predictor Model 2: Monthly Drinking Days** | | | |
| Real world comparison | Odds Ratio | Lower 95% CI | Upper 95% CI |
| 2 drinking days per month relative to 1 (median to LQ) | 1.10 | 1.06 | 1.14 |
| 7 drinking days per month relative to 2 (UQ to median) | 1.32 | 1.19 | 1.46 |
| 7 drinking days per month relative to 1 (UQ to LQ) | 1.45 | 1.26 | 1.66 |
| **Significant Predictor Model 2: Units per Occasion** | | | |
| Real world comparison | Odds Ratio | Lower 95% CI | Upper 95% CI |
| 8 units per drinking occasion relative to 2 (median to LQ) | 1.13 | 1.04 | 1.23 |
| 14 units per drinking occasion relative to 8 (UQ to median) | 1.07 | 1.02 | 1.12 |
| 14 units per drinking occasion relative to 2 (UQ to LQ) | 1.21 | 1.06 | 1.38 |
| 2 Pints per drinking occasion relative to 1 (4.6 units to 2.3) | 1.06 | 1.02 | 1.10 |
| 4 Pints per drinking occasion relative to 1 (9.2 units to 2.3) | 1.14 | 1.04 | 1.24 |
| 375ml bottle of spirits per drinking occasion relative to 1 pint (15 units to 2.3) | 1.21 | 1.06 | 1.38 |
| 2 bottles of wine per drinking occasion relative to 1 (19.5 units to 9.75) | 1.10 | 1.03 | 1.17 |

## **Evaluating the impact of Exclusions DUE to missing data**

A table was produced to demonstrate the number and rate of missing data across each of the predictor and outcome variables, which lead to the exclusion of adolescents’ treatments from the analysis. Due to low numbers at different points along the exclusion processing however, the vast majority of that table had to be suppressed, in line with government rules on suppressing data to prevent the potential identification of individuals. This table has therefore not been included, instead we present Supplementary Table 8, showing the total adolescents removed from each group due to missing data.

Supplementary Table 8: Rates of missing data across predictors and outcomes.

| **Group** | **Initial Count** | **Exclusions due to missing data** | **Final Count** | **Missing Data Rate** |
| --- | --- | --- | --- | --- |
| All Treatments | 2879 | 258 | 2621 | 9% |
| Female | 1810 | 170 | 1640 | 9% |
| Referral: Education | 1150 | 67 | 1083 | 6% |
| Referral: Criminal Justice | 322 | 37 | 285 | 11% |
| Referral: Self | 129 | 12 | 117 | 9% |
| Referral: Relative/Friend | ** | ** | 74 | ** |
| Referral: Social Care | 545 | 65 | 480 | 12% |
| Referral: Substance Misuse Service | ** | * | 41 | ** |
| Referral: Health Services | 521 | 53 | 468 | 10% |
| Referral: Other | 84 | 11 | 73 | 13% |
| Behavioural Disability | 43 | 0 | 43 | 0% |
| Early Use Onset | 2182 | 103 | 2079 | 5% |
| Illicit Substances | ** | * | 184 | ** |
| Anti-Social | 487 | 62 | 425 | 13% |
| Mental Health Need | 1132 | 106 | 1026 | 9% |
| Other's Substance Use | 572 | 66 | 506 | 12% |
| Domestic Abuse | 466 | 48 | 418 | 10% |
| NEET | 192 | 22 | 170 | 11% |
| Self-Harm | 756 | 69 | 687 | 9% |
| Looked After Child | 230 | 22 | 208 | 10% |
| Child in Need | 232 | 24 | 208 | 10% |
| Child Protection Plan | 171 | 23 | 148 | 13% |
| Sex Exploitation | 118 | 17 | 101 | 14% |
| Pregnant or Parent | 81 | 5 | 76 | 6% |
| Housing Problem | 138 | 0 | 138 | 0% |
| Financial Year of Exit 18/19 | 523 | 38 | 485 | 7% |
| Financial Year of Exit 19/20 | 609 | 65 | 544 | 11% |
| Financial Year of Exit 20/21 | 401 | 39 | 362 | 10% |
| Financial Year of Exit 21/22 | 603 | 45 | 558 | 7% |
| Financial Year of Exit 22/23 | 743 | 71 | 672 | 10% |
| Incomplete treatments | 315 | 52 | 263 | 17% |
| Complete treatments | 2564 | 206 | 2358 | 8% |

*Data supressed due to low count

**Data supressed to prevent inductive disclosure resulting from related supressed field.

A large number of incomplete treatments were reduced relative to the sample. Missing data affected two variables, related to a client’s “Treatment Outcomes Profile”. Adolescents were excluded from the main analysis when no TOP was found sufficiently close to their treatment start date to represent their drinking behaviour at treatment start, or when either one of the specific fields was missing. Unlike other variables, 9% (of the 17%) of incomplete treatments were removed specifically due to no TOP being identified rather than due to missing specific data *from* an identified TOP, which suggests adolescents abandoning treatments prior to a TOP being completed. Although this would be an interesting group in itself to examine, numbers are too small here to do so.

To understand the impact on missing data, the entire analysis was repeated, including all previously excluded cases and excluding the predictors “Monthly Drinking Days” and “Units per occasion”. The final Model results for incomplete vs. complete treatments are in Supplementary Table 9, H-LT χ² = 11.57, (d.f. = 8 *p* = 0.17). The Model for non-abstinent exits vs. abstinent exits can be seen in Supplementary Table 10, H-LT χ² = 3.25, (d.f. = 8 *p* = 0.92).

In each case, the stepwise selected variables were the same (Supplementary Table 9 and Table 10) with similar coefficients/odds ratios as the results presented in the main manuscript (Table 2 and Table 3), and we therefore conclude the exclusion of treatments as per Supplementary Table 8 does not affect the interpretation of our main analysis.

Supplementary Table 9: Summary of Model predicting incomplete vs complete treatments including excluded treatments (Alternate Model 1)

| **Predictor** | **Coefficient** | **Std. Error** | **Wald z** | ***p*** | **Odds Ratio (OR)** | **OR Lower CI** | **OR Upper CI** |
| --- | --- | --- | --- | --- | --- | --- | --- |
| (Intercept) | -4.47 | 0.37 | -12.17 | <0.001 |  |  |  |
| Age^#^ | 0.01 | 0.00 | 6.07 | <0.001 | 1.01 | 1.01 | 1.01 |
| Child Protection Plan | 0.68 | 0.21 | 3.17 | <0.01 | 1.97 | 1.29 | 2.99 |
| Financial Year of Exit 20/21 | 0.52 | 0.15 | 3.44 | <0.001 | 1.69 | 1.25 | 2.27 |
| NEET | 0.73 | 0.19 | 3.91 | <0.001 | 2.08 | 1.44 | 3.01 |

^#^Predictor transformed as (Age at treatment start)^2^

Table 10: Summary of Model predicting non-abstinent vs abstinent completions including excluded treatments (Alternate Model 2)

| **Predictor** | **Coefficient** | **Std. Error** | **Wald z** | ***p*** | **Odds Ratio (OR)** | **OR Lower CI** | **OR Upper CI** |
| --- | --- | --- | --- | --- | --- | --- | --- |
| (Intercept) | -3.82 | 0.31 | -12.39 | <0.001 |  |  |  |
| Age^#^ | 0.01 | 0.00 | 10.84 | <0.001 | 1.01 | 1.01 | 1.01 |
| Early Onset Use | 0.48 | 0.11 | 4.33 | <0.001 | 1.62 | 1.30 | 2.01 |
| Illicit Substances | 0.77 | 0.17 | 4.40 | <0.001 | 2.15 | 1.53 | 3.03 |
| Mental Health Need | 0.36 | 0.09 | 4.13 | <0.001 | 1.43 | 1.21 | 1.70 |
| Other’s Substance Use | 0.30 | 0.11 | 2.81 | <0.01 | 1.35 | 1.09 | 1.66 |

^#^Predictor transformed as (Age at treatment start)^2^

# ****References****

Fox, J., & Weisberg, S. (2019). An R Companion to Applied Regression. In (Vol. Third edition): Sage, Thousand Oaks CA.

Hartig, F. (2022). DHARMa: Residual Diagnostics for Hierarchical (Multi-Level / Mixed) Regression Models. In. R package version 0.4.6.

*IMB Corp. Released 2023. IBM SPSS Statistics for Windows, Version 29.0.2.0 Armonk, NY: IBM Corp*. In.

NHS England. *National Drug Treatment Monitoring System*. Retrieved June 2024 from <https://digital.nhs.uk/data-and-information/information-standards/information-standards-and-data-collections-including-extractions/publications-and-notifications/standards-and-collections/dcb0107-national-drug-treatment-monitoring-system> .

Office for Health & Improvement Disparities. (2023). Reference Data (Core Dataset Q). In. National Drug Treatment Monitoring System: National Drug Treatment Monitoring System.

Posit team. (2024). In *RStudio: Integrated Development Environment for R. Posit Software, PBC, Boston, MA. URL* [*http://www.posit.co/*](http://www.posit.co/).

R Core Team. (2024). *R A Language and Environment for Statistical Computing*. In R Foundation for Statistical Computing, Vienna, Austria.

Vanegas, L., Rondón, L., & Paula, G. (2024). glmtoolbox: Set of Tools to Data Analysis using Generalized Linear Models (R package version 0.1.1). In.

Wickham, H. (2007). Reshaping data the reshape package. In: Journal of Statistical Software.

Wickham, H. (2016). ggplot2 Elegant Graphics for Data Anlaysis. In: Springer-Verlag New York.

Wickham, H., François, R., Henry, L., Müller, K., & Vaughan, D. (2024). dplyr: A Grammar of Data Manipulation. In.

Wickham, H., Hester, J., & Bryan, J. (2021). readr: Read Rectangular Text Data. In *R package version 2.1.5,* [*https://github.com/tidyverse/readr*](https://github.com/tidyverse/readr)*,* [*https://readr.tidyverse.org*](https://readr.tidyverse.org) *.*

Wickham, H., Pedersen, T., & Seidel, D. (2023). scales: Scale Functions for Visualization (R package version 1.3.0). In.
